# From subjective assessment to data-driven diagnosis: the AI revolution in multimodal early detection of Sjögren’s disease

**DOI:** 10.3389/fimmu.2026.1792331

**Published:** 2026-07-30

**Authors:** Yixuan Zhou, Bingbing Yu, Rong Chang, Yaya Ding, Yuan Wang, Man Han

**Affiliations:** 1 Department of Rheumatology, The First Affiliated Hospital of Anhui University of Chinese Medicine, Hefei, Anhui, China; 2 Department of Rheumatology, Guang’anmen Hospital China Academy of Chinese Medical Sciences, Beijing, China; 3 Department of Traditional Chinese Medicine, Qinghai University Medical College, Xining, China; 4 Key Laboratory of Xin’an Medicine, Ministry of Education, Anhui University of Chinese Medicine, Hefei, Anhui, China

**Keywords:** artificial intelligence, deep learning, early diagnosis, machine learning, precision medicine, Sjögren’s disease

## Abstract

Sjögren’s disease (SjD), a chronic autoimmune disorder affecting exocrine glands, faces significant diagnostic challenges due to its highly heterogeneous symptoms, subjective interpretation of imaging findings, and reliance on invasive biopsies, often resulting in delayed diagnosis by 3–7 years. This review posits that the integration of diverse data streams—from medical imaging to molecular omics—is the pivotal key to overcoming diagnostic heterogeneity, and that AI serves as the indispensable engine to power this integration. We systematically synthesize evidence showing that deep learning models, such as Fully Convolutional Dense Network (FCN-DenseNet), can automate salivary gland ultrasound segmentation; computed tomography (CT)-based AlexNet may achieve diagnostic performance comparable to radiologists; and AI-driven analyses of pathology, metabolomics, and genomics have revealed potential biomarker patterns, including metabolomic and immune-clustering models with reported AUC values of 1.00. However, these findings should be interpreted cautiously because many studies were conducted in small, retrospective, single-center cohorts or relied mainly on internal validation, limiting their clinical robustness and generalizability. We thus critically advocate for a future paradigm centered on causally-aware, multimodal fusion frameworks—moving beyond mere correlation to model disease mechanisms—and the adoption of federated learning to navigate data privacy while leveraging large-scale genomics. The forthcoming challenge is not merely technical but conceptual: to develop interpretable AI that can deconstruct SjD into mechanistically defined subtypes, thereby bridging the critical gap between algorithmic prowess and clinically actionable insights for definitive early intervention and personalized treatment strategies.

## 1 Introduction

Sjögren’s disease (SjD), a chronic inflammatory autoimmune disorder affecting multiple exocrine glands, is a global condition with cases reported worldwide across diverse regions, ethnicities, and populations. Primarily targeting salivary and lacrimal glands, its hallmark symptoms include persistent dry mouth and eyes. In China, the prevalence ranges from 0.3% to 0.7% (1), predominantly affecting women aged 40–60 with a male-to-female ratio of 1:9 (2). Beyond these core symptoms, patients often experience fatigue and joint pain, severely impairing daily activities like eating and vision, which drastically reduces quality of life. Severe cases may develop complications such as pulmonary fibrosis and lymphoma, requiring prolonged multidisciplinary care and consuming substantial medical resources. This not only causes significant physical and emotional distress but also imposes substantial burdens on personal health, family finances, and the healthcare system.

Currently, lip gland biopsy remains the gold standard pathological criterion for diagnosing Sjögren’s disease (3). The diagnosis is established by evaluating the extent of focal lymphocytic infiltration within the gland (focal score ≥1/4 mm²), which offers advantages such as high objectivity and reproducibility. Both the 2002 International Classification and the 2016 American College of Rheumatology/European Union of Rheumatology (ACR/EULAR) classification standards list pathological examination results as core diagnostic criteria (4). However, early diagnosis of Sjögren’s disease faces multiple challenges: On one hand, the disease presents with complex and varied symptoms, with some patients only showing mild discomfort that can be easily confused with other common causes of dry mouth and eyes. On the other hand, traditional imaging examinations exhibit strong subjectivity, with limitations such as high operator dependence and significant subjective interpretation biases, making it difficult for clinicians to detect subtle glandular structural changes early, thereby exacerbating diagnostic delays.

In recent years, the introduction of artificial intelligence (AI) technology has opened new pathways to overcome the aforementioned bottlenecks. The heterogeneity in clinical manifestations and pathophysiology of Sjögren’s disease—ranging from varying symptom severity and significant differences in glandular damage to multi-pathway immune-inflammatory mechanisms—poses challenges not only for early diagnosis but also for developing effective therapies. Deep learning-based imaging omics technology, however, can automatically extract thousands of quantitative features from routine medical imaging and combine machine learning (ML) algorithms (such as convolutional neural networks and random forests) to build predictive models, enabling objective quantification of glandular damage. For instance, integrating salivary gland ultrasound (SGUS) imaging omics features with serum autoantibody levels (anti-SSA/Ro antibodies, anti-SSB/La antibodies) allows AI to improve early glandular lesion detection accuracy by over 30%, providing technical support for identifying disease diagnosis windows (5). This multimodal data fusion strategy not only compensates for the subjectivity of traditional imaging examinations but also reveals potential correlations between imaging features, pathological biopsies, and molecular markers, offering precise decision-making foundations for personalized Sjögren’s disease diagnosis and treatment. The approach demonstrates significant clinical translational value.

As global understanding of Sjögren’s disease deepens, its status as a major burden of chronic autoimmune diseases has garnered significant attention. Current research indicates that patients with Sjögren’s disease exhibit higher rates of disability and complication-related mortality compared to the general population. Persistent symptoms often lead to psychological issues such as anxiety and depression, further exacerbating the disease’s negative impact on health. Additionally, delayed diagnosis and lack of targeted treatments result in prolonged medication regimens, organ monitoring, and rehabilitation interventions for advanced-stage patients, significantly increasing healthcare costs and straining global medical resources. In this context, the application of artificial intelligence in Sjögren’s disease diagnosis and treatment not only provides technical solutions for early detection but also opens new avenues for optimizing therapeutic strategies and reducing disease burden. Future multi-center studies are needed to validate its clinical value and facilitate the efficient transition of this technology from laboratory to clinical practice.

## 2 AI-assisted clinical assessment and stratification

The clinical diagnosis and patient stratification of Sjögren’s disease are undergoing profound transformations driven by intelligent technologies. Recent breakthroughs in attention-based deep learning (DL) models have enabled automated evaluation of lesions (FS≥1) through systematic analysis of whole-slide images (WSI) from 271 lip salivary gland biopsy specimens. The model demonstrated promising diagnostic performance: AUC of 0.932(95%CI:0.881-0.984, sensitivity of 87%, specificity of 84%, and accuracy of 85.2% (6). This achievement substantially outperforms traditional manual assessment methods while effectively eliminating observer bias, thereby establishing a standardized technical platform for SjD diagnosis and biomarker discovery. Concurrently, a research team from the University of Florida achieved a major breakthrough in pediatric SjD stratification. By ingeniously applying latent variable analysis combined with advanced algorithms including random forests, gradient boosting decision trees, and artificial neural networks, they precisely categorized suspected SjD pediatric patients into three distinct groups: Class I (predominantly dry symptoms with positive detection), Class II (obvious symptoms with negative detection), and Class III (mild symptoms with negative detection). Researchers further developed the Florida Score system through causal graph modeling, which has proven highly potentially clinically applicable in assisting precise classification and long-term monitoring of suspected pediatric SjD patients (7).

In the practical application of automated machine learning platforms, a study involving 86 suspected Sjögren’s disease patients demonstrated the tremendous potential of technological breakthroughs. The research team meticulously constructed a large database containing 172 tissue sections, with 74 meeting SjD classification criteria (FS≥1) and 98 being normal salivary gland tissues. By employing a convolutional neural network model based on the Residual Network-152 (ResNet-152) architecture, the system achieved high sensitivity of 89.47% and excellent specificity of 88.24%, providing reliable technical support for objective disease differentiation (8). This technological advancement carries dual significance: enhancing diagnostic standardization and providing clinicians with robust decision support systems. However, comparative analysis between deep learning and traditional ML methods revealed some noteworthy issues. Although DL excels in medical imaging diagnosis, it tends to overfit on small sample datasets. A large-scale systematic review and meta-analysis showed that DL algorithms match the overall diagnostic performance of medical professionals, with combined sensitivity of 87.0% and 86.4%, and specificity of 92.5% and 90.5% respectively (9).

From the perspective of algorithm selection and performance optimization, current research demonstrates clear preference trends. A systematic analysis of 48 disease prediction studies reveals that Support Vector Machines (SVM) emerged as the most frequently used supervised learning algorithm (29 studies), followed by Naive Bayes algorithm (23 studies) (10). This preference reflects machine learning’s unique advantages in medical diagnosis: its ability to efficiently process large-scale, high-dimensional data and identify complex patterns that traditional statistical methods struggle to detect. In the critical field of patient stratification, multi-omics data-based clustering analysis has demonstrated unique clinical value. Recent studies have identified three molecular subpopulations with significantly different immune cell infiltration patterns in SjD patients, which can be precisely identified using random forest models (AUC = 1.00) (11). The reported AUC of 1.00 should therefore be interpreted cautiously, as it may reflect performance within a controlled clustering framework rather than stable classification accuracy in broader clinical cohorts. Nevertheless, these molecular-clustering findings provide useful preliminary evidence for immune subtyping and personalized treatment strategies. For clinical translation, further validation is needed in prospective, multicenter, large-cohort studies that have predefined diagnostic endpoints.

## 3 Innovation in AI-driven imaging diagnosis

The diagnostic technology of salivary gland ultrasound is undergoing a critical transformation characterized by optimized scoring systems and deep integration of intelligent technologies. A landmark multicenter study conducted by Zhang et al. in 2021 established new benchmarks in this field. Through rigorous scientific design, the study validated the superior diagnostic efficacy of the Outcome Measures in Rheumatology Clinical Trials (OMERACT) four-grade scoring system (0–3 points). The system achieved 75.6%-77.2% sensitivity and 91.6%-92.2% specificity with evaluations of unilateral parotid and submandibular glands (total score ≥4 points), demonstrating an area under the curve (AUC) of 0.904-0.910, which is comparable to the traditional four-gland total score assessment (AUC = 0.908) (12). This significant finding substantially enhanced the practicality and clinical applicability of the scoring system. Subsequent research by Tang et al. in 2023 further confirmed the excellent generalization performance of the OMERACT scoring system, showing an AUC of 0.828 (sensitivity 75.9%, specificity 89.7%) when the four-gland total score was ≥8 points, highly consistent with Zhang et al.’s findings (13).

In the intelligent segmentation and scoring of submandibular gland images, deep learning models have demonstrated remarkable technical advantages. The FCN-DenseNet model achieved a breakthrough in salivary gland segmentation, with an Intersection-Union (IoU) score of 0.85. This not only surpassed the observer agreement among clinical experts (IoU=0.76) but also processed 24.5 frames per second, achieving a perfect balance between high speed and accuracy (8). Kise et al. compared the performance of deep learning systems and inexperienced radiologists in diagnosing SGUS images from 200 patients. The DL system outperformed human interpretation in accuracy, sensitivity, and specificity (89.5%,90.0%,89.0%) (14). Similar results were observed in submandibular gland detection, where the DL system outperformed human diagnosis in all three metrics. Another study using 600 SGUS images extracted 907 imaging omics features and employed a genetic algorithm to select the optimal feature subset. This approach achieved a Cohen’s Kappa coefficient (κ) of 0.7 for the multi-layer perceptron classifier, surpassing both the average scoring consistency of clinical doctors (κ=0.67) and approaching the ideal level of intra-observer consistency (κ=0.71) (15). These studies demonstrate that deep learning addresses the limitations of inexperienced physicians in image interpretation while delivering preliminary technical support for the standardization and automation of SGUS scoring.

Despite these promising advances, the broader validation and standardization of such AI models remain formidable challenges. A large-scale systematic review specifically targeting DL in radiology diagnosis revealed a concerning trend: Among all external validation studies, 81% showed varying degrees of performance decline on external datasets, with 49% experiencing moderate deterioration and 24% demonstrating statistically significant deterioration (16). To address this critical challenge, the SGUS reliability study under the European multicenter HarmonicSS project laid crucial groundwork for establishing a standardized scoring system. The project brought together 20 ultrasound specialists from nine European countries, who conducted reliability validation of the De Vita scoring system and OMERACT scoring system through specialized atlas guidance and systematic training. Results demonstrated near-perfect intra-assessor reliability (Light’s kappa values of 0.86 and 0.87) and good inter-assessor reliability (Light’s kappa values of 0.75 and 0.77) for both scoring systems, with no significant differences observed among ultrasound physicians of varying experience levels. This confirmed that adequately trained operators can achieve reliable assessment outcomes (17).

Building on standardized protocols, comparative analysis of diagnostic performance across different SGUS scoring systems became a focal point. A comprehensive systematic review and meta-analysis incorporating 24 high-quality studies involving over 3,000 patients provided valuable evidence-based medical insights. The findings revealed: the 0–4 point scoring system achieved combined sensitivity of 75% (I²=92.0%) and specificity of 93% (I²=71.5%), with a diagnostic odds ratio of 71.26; the 0–16 point scoring system demonstrated combined sensitivity of 84% and specificity of 88%; while the 0–48 point scoring system showed combined sensitivity of 75% and specificity of 95%. Comprehensive analysis indicates that the 0-4-point scoring system demonstrates superior performance in controlling specificity and heterogeneity, making it a more suitable universal diagnostic criterion for SGUS (1).

In the field of Computed Tomography (CT) imaging diagnosis for Sjögren’s disease, the introduction of intelligent technologies has achieved a potential improvement in diagnostic efficiency. This study introduced deep learning models into the CT imaging diagnosis of SjD. Based on CT image data from 25 confirmed patients and 25 control subjects, the system achieved a diagnostic accuracy rate of 96.0%, 100% sensitivity, and 92.0% specificity. However, this result was derived from a preliminary dataset of only 25 SjD patients and 25 controls. The reported 100% sensitivity may therefore reflect performance under controlled experimental conditions rather than stable diagnostic accuracy in broader clinical settings. The results showed that the DL system’s diagnostic performance was comparable to that of experienced radiologists (accuracy 98.3%), but significantly outperformed inexperienced physicians (accuracy 83.5%), indicating the system’s potential to provide effective support for CT imaging diagnosis (18). Another significant study ingeniously combined radiomics and machine learning techniques, constructing an efficient SjD diagnostic prediction model through analysis of 132 parotid CT images from 66 subjects. The research team extracted 129 carefully selected radiomics features using the 3DSlicer platform, covering multiple dimensions of first-order, shape, and texture characteristics, and automated model generation was achieved through the PredictionOne platform. The model demonstrated favorable performance in internal validation (AUC = 0.92, accuracy 0.88), with time-validation further confirming its high performance (AUC = 0.96, recall rate 0.92) (19).

In the field of magnetic resonance imaging (MRI) imaging diagnosis for SjD, the deep mining of radiomics features has opened new technical pathways for early disease identification. A well-designed study involving 164 patients extracted up to 1,548 texture features from T1-weighted and fat-suppressed T2-weighted images, constructing a clinically valuable radiomics score (Radscore). Results showed that SjD patients exhibited significantly higher Radscore values than healthy controls (p<0.001), providing quantitative evidence for precise differential diagnosis. Among five ML models, the Extreme Tree Classifier (ETC) stood out with a test set AUC of 0.87, demonstrating exceptional capability in identifying early parotid gland damage (20). However, this single-center retrospective study with 164 patients requires external validation in larger cohorts.

Despite remarkable technological advancements, bias control and clinical validation remain critical challenges limiting the widespread application of AI imaging diagnostic systems. In-depth research indicates that medical imaging AI systems may exhibit various types of biases, including data acquisition bias, measurement bias, and deployment bias (21, 22). These biases often stem from variations in imaging equipment, software versions, and acquisition parameters among different manufacturers. An exploratory study on chest X-ray pneumonia detection revealed that AI models could accurately predict the source hospital of images, strongly suggesting that models might learn hospital-specific characteristics unrelated to disease diagnosis (23). To systematically address these technical challenges, researchers have proposed various innovative external validation strategies, including geographic validation, temporal validation, and technical validation (24, 25). These multidimensional validation methods help comprehensively evaluate the generalization ability and stability of AI systems in complex real-world environments.

In terms of strict clinical evaluation requirements, randomized controlled trials (RCTs) have become increasingly crucial. A comprehensive systematic review found that 70% of RCTs specifically assessing the clinical effectiveness of AI demonstrated positive primary endpoints, primarily involving significant improvements in diagnostic output or performance metrics (26). However, the reality remains less than ideal: most trials are still limited to single-center designs with insufficient demographic reporting, severely restricting the generalizability and clinical applicability of research findings. Nevertheless, some successful multi-center validation cases provide valuable references for the future development of SjD imaging diagnosis. The International Ovarian Tumor Machine Learning Collaborative Research Project stands as a prime example, successfully aggregating 17,119 ultrasound images from 20 medical centers across 8 countries. The transformer model trained using a leave-one-center cross-validation scheme demonstrated satisfactory stability across multiple centers, different ultrasound systems, and various patient age groups (27). This large-scale multi-center validation model provides crucial methodological references and practical guidance for the successful clinical translation of medical AI technologies.

## 4 AI-assisted pathology and biomarkers

The field of pathological and histological analysis in Sjögren’s disease is undergoing profound transformations driven by intelligent technologies. These pioneering innovations are continuously breaking through the inherent limitations of traditional diagnostic methods, providing unprecedented technical tools for precise disease differentiation and risk prediction. In the core task of accurately distinguishing SjD from non-SjD cases, the innovative application of automated machine learning platforms has achieved remarkable progress. A landmark study conducted in-depth analysis on 172 salivary gland biopsy slides from 86 SjD patients using this platform demonstrated exceptional performance of a convolutional neural network model based on the ResNet-152 architecture, achieving 89.47% high sensitivity and 88.24% excellent specificity (28). This technological breakthrough furnishes reliable tools for objective disease differentiation and substantially mitigates the inevitable individual variations inherent in manual diagnosis. By accurately capturing subtle features in pathological slides through deep learning techniques, it substantially enhances the overall accuracy of differential diagnosis. The widespread successful application of ML in disease diagnosis has also laid a solid theoretical foundation and technical support for SjD pathological analysis.

A comprehensive analysis of 1,216 related papers in Scopus and Web of Science databases reveals that ML technology holds tremendous potential in disease diagnosis. Facing the complex challenges of diverse disease mechanisms and symptoms, machine learning-based disease diagnosis has made remarkable progress in algorithm optimization, disease type identification, data processing, and evaluation metrics. These advancements provide crucial technical support and methodological guidance for applying artificial intelligence in early disease detection (29). A multicenter study published in The Lancet Rheumatology in 2025 developed a DL model based on the Clustering-constrained Attention Multiple-instance learning (CLAM) architecture for the automatic classification of focus scores (FS) and diagnosis of Sjögren’s disease. External validation showed that the area under the receiver operating characteristic curve (AUROC) was 0.88 (95% CI: 0.82-0.94) for FS classification and 0.89 (95% CI: 0.82-0.94) for SjD diagnosis, with even better performance in anti-SSA-negative patients (AUROC = 0.92). This study applied heatmap visualization, an interpretable technology, to SjD pathological diagnosis, enabling pathologists to intuitively understand the AI decision-making process. This “white-box” approach significantly enhances the credibility of clinical applications and provides important multicenter validation evidence for the clinical translation of AI-assisted pathological diagnosis (30).

In the innovative development of pathological analysis frameworks, the Cell-Tissue Atlas Pathological Analysis Model (CTG-PAM) proposed by a multicenter study represents a major technological breakthrough in this field. This innovative model breaks free from the limitations of traditional methods confined to single-cell or tissue morphology observation, adopting a systematic analytical approach that considers single-cell characteristics, intercellular interactions, and the complex network of cell-tissue relationships. Notably, the model achieved an excellent AUC value of 0.8035 on external test datasets, with sensitivity and accuracy reaching 98.21% and 93.75% respectively, significantly outperforming traditional deep learning methods (31). This global analytical capability of pathological microenvironments provides a novel technical perspective and methodological framework for revealing the complex pathological mechanisms of SjD and comprehensively improving diagnostic efficacy. In the frontier field of disease risk prediction, AI applications based on whole-slide images from lip gland biopsies extend the clinical value of advanced technologies to broader practical applications. The research team utilized pre-trained models such as ResNet50, InceptionV3, and EfficientNet-B5 to extract image features from WSI data of 221 SjD patients, and constructed a high-risk extraglandular organ involvement (HR-OI) prediction model by integrating multi-instance learning and ensemble learning techniques. The integrated model demonstrated superior ROC AUC values on both internal and external validation datasets, effectively identifying key pathological features associated with HR-OI risk. This provides objective evidence for clinical decision-making and personalized treatment strategies in SjD patients (32).

In the crucial field of detection efficiency optimization, the enhanced You Only Look Once version 8 (YOLOv8) model has demonstrated remarkable technical breakthroughs. By innovatively incorporating advanced components such as multi-dimensional attention modules and S-MPDIoU loss function, this model achieved a significant 9.1% improvement in lymphatic infiltration detection recall rate, with mAP.5 and mAP.95 metrics increasing by 3.2% and 2% respectively. This optimization delivers two critical improvements: a substantial reduction in the time cost of pathological analysis and the creation of an efficient, reliable platform to support rapid SjD diagnosis and dynamic disease assessment (33). In the emerging field of SjD metabolomics research, systematic mining of multi-dimensional biomarkers has opened new quantitative analytical pathways for disease diagnosis. Breakthroughs in serum metabolomics analysis are particularly noteworthy. Researchers employed advanced Ultra-Performance Liquid Chromatography-High-Resolution Mass Spectrometry (UPLC-HRMS) technology to conduct in-depth comparative analysis of serum metabolomes between SjD patients and healthy controls. By leveraging advanced algorithms like Least Absolute Shrinkage and Selection Operator (LASSO), random forest, and Extreme Gradient Boosting (XGBoost), the team successfully identified 21 significantly different metabolites closely associated with amino acid and lipid metabolic pathways. Notably, 2-hydroxypropionic acid, L-carnitine, and cyclic AMP were identified as potential biomarkers for SjD, with diagnostic efficacy (AUC = 1.00) significantly outperforming traditional methods (34).

Metabolomic studies of diverse biological specimens have opened up promising new technical pathways for non-invasive disease diagnosis. Salivary metabolomics research has revealed a series of significant findings: Patients with Sjögren’s disease exhibit markedly elevated levels of oxidative stress-related metabolites (such as lactic acid and alanine) and T-cell proliferation-associated amino acids (like arginine and leucine) in saliva, providing valuable biological clues for convenient non-invasive diagnosis (35). Similarly, lacrimal metabolomics research has achieved major breakthroughs, successfully identifying 9 key metabolites (including serine, aspartic acid, dopamine, and 6 lipid molecules). These molecular markers effectively distinguish SjD from other causes of dry eye, achieving an excellent ROC-AUC of 0.83 (36). These diagnostic characteristics remain unaffected by age, gender, or anti-SSA antibody status, demonstrating strong clinical applicability. Analysis of local tissue metabolic features provides more refined technical tools for precise disease evaluation. Lipid gland metabolomics research, through the combined application of elastic network regularization Logit model and random forest algorithm, identified unique diagnostic features composed of kynurenine and 5 phospholipids, achieving an excellent ROC-AUC of 0.86. These features also showed significant correlations with lymphocyte infiltration levels and anti-SSA antibody levels, providing crucial technical references for accurate assessment of local lesions (37).

In the cutting-edge research of SjD genes and immune characteristics, the deep application of intelligent technologies is providing unprecedented breakthroughs in key technologies for deciphering complex disease mechanisms and optimizing precision diagnosis and treatment strategies. The precise analysis of gene regulatory networks is considered the core technical link for deeply understanding the pathogenesis of SjD, while the innovative combination of machine learning and Weighted Gene Co-expression Network Analysis (WGCNA) provides powerful technical tools for this critical task. Through WGCNA analysis of two public datasets (GSE40568 and GSE40611), the research team successfully identified 376 differentially expressed genes (DEGs) in primary Sjögren’s syndrome patients, with SAMD9 and IFIT3 confirmed as key genes with outstanding diagnostic potential. The study revealed that SAMD9 is closely associated with immune cell infiltration, and constructed a competing endogenous RNA (ceRNA) regulatory network containing 2 key genes, 12 miRNAs, and 124 lncRNAs, providing new research directions for elucidating the molecular mechanisms and potential therapeutic targets of SjD (38). This precise ability to capture complex gene regulatory relationships far surpasses the technical limitations of traditional single-gene analysis methods, laying a solid theoretical foundation for subsequent in-depth research at the molecular level.

Technological advancements in immune heterogeneity analysis are equally remarkable, with intelligent technologies further demonstrating unique technical value in this field. Recent studies have identified three molecular subpopulations with distinct immune cell infiltration patterns in SjD patients. Using a random forest model, these subpopulations can be precisely identified and accurately distinguished (AUC = 1.00) (11). This breakthrough provides crucial scientific evidence and technical support for precise immunotyping and the development of personalized treatment strategies.

Multi-omics integration studies have leveraged the analytical prowess of machine learning to uncover intricate correlations between gene expression patterns and disease phenotypes. In a landmark study of primary Sjögren’s syndrome patients, researchers identified a significant association between interferon-stimulated genes (ISGs) and anti-SSA/Ro60 antibodies. Five key genes—EPSTI1, EIF2AK2, and others—were screened and found to show strong correlations with disease activity (ESSD AI) and CD45 levels (39). Another breakthrough study ingeniously combined WGCNA with six ML algorithms, successfully identifying five core hub genes (OAS1, EIF2AK2, etc.) enriched in the Type I interferon signaling pathway. These genes are closely linked to the activation of critical immune cells like CD8+ T cells and NK cells (40). These findings deepen our scientific understanding of the complex immune mechanisms in SjD, signaling a paradigm shift in clinical management from a traditional homogeneous framework toward a new era of precision subtyping.

The exceptional analytical capabilities of intelligent technologies have also opened new possibilities for expanding the academic boundaries of SjD research. Through machine learning, researchers have uncovered shared diagnostic genes (CSF2RB, CXCR4, and LYN) between periodontitis (PD) and SjD, with these shared genetic markers demonstrating outstanding diagnostic performance (AUC> 0.85) (41). Recent research on epigenetic regulatory mechanisms has opened up new technical directions for innovative immunotherapy strategies. Through in-depth studies of m6A methylation regulators based on the expression patterns of eight key factors, researchers successfully classified SjD patients into two distinct subtypes with unique immune characteristics (42). The discovery of cross-disease diagnostic biomarkers holds immense clinical value and translational potential. The unique interferon signature of anti-SSA/Ro60-positive patients has been successfully identified, while the expression profiles of three key genes (ATP10A, MX1, and others) demonstrate promise as universal diagnostic markers for autoimmune diseases including SjD and systemic lupus erythematosus (43). This breakthrough provides novel academic perspectives and technical pathways for studying the pathogenic associations of autoimmune disorders.

The complete translational pipeline from target discovery to drug screening demonstrates the immense potential of artificial intelligence in precision medicine. Building upon the identification of key gene targets, the research team employed molecular docking technology to conduct systematic drug repurposing studies. Using a computer-aided drug design platform, researchers evaluated the binding capabilities of numerous small molecules with hub gene proteins, successfully identifying three potential therapeutic compounds—DB08006, DB08036, and DB15308—that exhibited favorable docking scores for both ELAVL1 and IGF1R. This AI-driven drug discovery strategy not only significantly shortened traditional drug development timelines but, more importantly, provided novel therapeutic candidates for treating SjD, laying a solid foundation for subsequent pharmacological validation and clinical trials (44).

In the research field of biomarker screening and therapeutic target discovery with significant translational value, multidimensional machine learning analysis has successfully bridged the gap between basic scientific research and clinical applications. Breakthroughs in gene expression profiling and drug discovery have been particularly noteworthy. A multi-omics study involving 1,341 samples, integrating transcriptome sequencing, microarray data, and ML methods, investigated the correlation between interferon-stimulated genes and anti-SSA/Ro 60 antibodies in SjD, identifying five key diagnostic genes—EPSTI1, EIF2AK2, IFI44, RSAD2, and OAS2—closely associated with disease severity and pathological scoring (39). The discovery of novel pathological mechanism biomarkers has provided fresh scientific perspectives for understanding disease mechanisms. Deep machine learning analysis revealed significant ferroptosis-related gene expression patterns in SjD patients. By integrating data from GEO and FerrDb databases, six ferroptosis-related genes—including TBK1 and SLC1A4—were identified as reliable diagnostic markers (45). The precise identification of tissue-specific biomarkers has provided crucial technical tools for advancing precision diagnostic technologies.

Lipidomics studies of canine salivary glands, leveraging the analytical capabilities of ML, successfully identified unique diagnostic signatures composed of kynurenine and five specific phospholipid molecules (AUC = 0.86), with phospholipid components showing significant correlation with lymphocyte infiltration severity (37). This systematic multi-omics data integration approach not only effectively overcomes the inherent technical limitations of single detection methods but also establishes a critical bridge from laboratory research to clinical application for precision diagnosis and targeted therapy strategies in SjD. This breakthrough significantly propels the entire research field toward more clinically relevant and translational value-oriented developments.

## 5 Discussion

**Figure 1:**
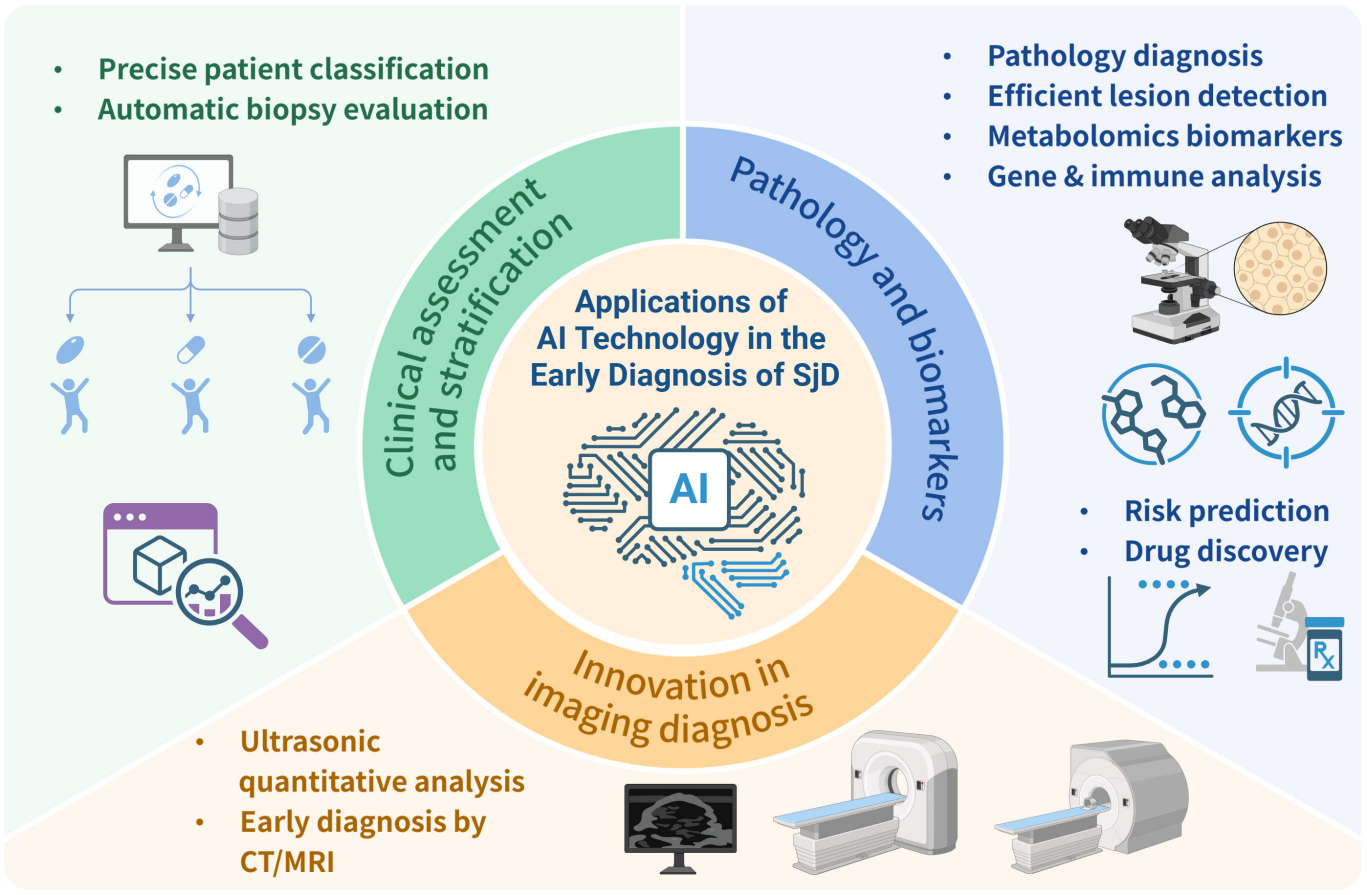
A panoramic view of core applications of AI technology in the early diagnosis of SjD. Created in BioRender.

This comprehensive review systematically examines the multidimensional applications of artificial intelligence in early-stage Sjögren’s disease diagnosis, covering technological breakthroughs across five core domains: clinical scoring, imaging diagnostics, pathological analysis, metabolomics, and genomics (**Figure 1**). Research findings demonstrate that AI is fundamentally transforming the diagnostic approach for this complex autoimmune disorder, shifting from traditional subjective assessments to objective, standardized intelligent diagnostic systems. Through systematic analysis of various technical pathways for early Sjögren’s disease detection, AI has shown significant potential in enhancing diagnostic accuracy, reducing diagnostic time, and achieving precise disease classification. Breakthroughs in AI technology for Sjögren’s disease diagnosis and treatment demonstrate remarkable characteristics of cross-disciplinary collaboration and mutual reinforcement. From intelligent innovations in imaging to objective advancements in pathology, and deep integration of multi-omics data, a comprehensive, multi-level intelligent diagnostic system has been established. In imaging, deep learning has achieved remarkable accuracy in automatic segmentation of salivary gland ultrasounds, providing objective and consistent gland volume measurements and echogenicity quantification, offering doctors timely pathological assessment references. Radiomics-based models extract hundreds of quantitative features from CT/MRI scans for grading glandular damage and distinguishing Sjögren’s disease subtypes. In pathology, attention-based neural networks and improved object detection algorithms not only automatically calculate focal intensity but also identify microscopic patterns like lymphatic infiltration, acinar destruction, and fibrosis across entire histological sections, achieving sensitivity and specificity of approximately 0.73 with strong generalization through external validation. Additionally, machine learning applications in metabolomics and transcriptomics data have revealed potential biomarkers such as 2-hydroxypropionic acid, L-carnitine, and EPSTI1, demonstrating AI’s ability to uncover pathological mechanisms that traditional methods struggle to detect. These breakthroughs across different technological domains are not isolated but rather complementary and synergistic. Imaging data on glandular structure and function validate pathological findings of cellular morphology, while metabolomics-derived biomarkers and genomic discoveries of molecular mechanisms form a complete pathogenic chain. The fusion analysis of multimodal data not only enhances the accuracy of single diagnostic methods but, more importantly, provides comprehensive scientific evidence for SS’s precise classification and personalized treatment strategies, spanning from molecular to cellular levels and from local to systemic perspectives. This marks the transition of SjD diagnosis and treatment from the traditional “unified approach” to the modern “precision classification” model, laying a solid technical foundation for achieving early identification, targeted intervention, and individualized therapy.

Despite these advances, several highly performing models, including CT-based DL models, serum metabolomic classifiers, immune-clustering models, and selected pathology-based systems, remain insufficiently validated for broad clinical implementation. Much of the current evidence is derived from small, retrospective, single-center cohorts or studies relying mainly on internal validation. Potential overfitting, feature-selection bias, domain shift, and insufficient calibration may lead to overestimated performance. Numerous studies perform feature selection and hyperparameter tuning on the full dataset before only reporting the optimal model. This practice results in overestimated predictive performance and ambiguous practical value for clinical diagnosis. Rigorous prospective external validation, together with comprehensive reporting fully compliant with the Transparent Reporting of a multivariable prediction model for Individual Prognosis Or Diagnosis-AI (TRIPOD-AI) guidelines, constitutes a prerequisite for reliably evaluating model performance. Therefore, these models should currently be regarded as promising decision-support approaches. Specifically, existing evidence-based and methodological research reveals that medical imaging AI frequently suffers performance degradation due to “domain shift” (differences in population composition, acquisition protocols, and workflow variations). The “scenario-specific adaptation” achieved through single-center optimization proves difficult to replicate across institutions, a risk equally applicable to SGUS, CT/MRI, and pathological tasks discussed in this paper (46). Feature stability in imaging remains a critical bottleneck for translational applications. Technical factors such as CT reconstruction kernels, slice thickness, and radiation dose can significantly alter radiomics feature distributions, with only a few features demonstrating reproducibility across different scans and parameters, directly constraining model robustness and cross-center consistency (47). For SGUS, even with standardized scoring frameworks, operator training levels, equipment, and preset parameters can influence interpretation consistency and the texture signals learned by algorithms. This highlights the need for further parameter calibration and reading consistency control before conducting external validation (48). In annotation and evaluation processes, caution is required regarding label noise and calibration inadequacies. Lip gland biopsies and whole-slide imaging (WSI) often employ weak annotations or coarse-grained labels, which can introduce observer bias and noise accumulation, thereby inflating apparent AUC while reducing true transferability. Recent evidence on pathological weak annotation denoising and robust learning demonstrates that even in high-performance scenarios, label quality and learning strategies remain critical determinants of external generalization limits (49). Furthermore, medical imaging deep learning models frequently suffer from poor probabilistic calibration. Unreported or uncorrected calibration issues may compromise risk threshold setting and clinical adoption (50). Meanwhile, positive findings dominate the literature, whereas null and negative results are rarely reported, despite being equally critical for accurately assessing model performance and clinical applicability.

From the laboratory breakthroughs of AI technology to its translation into practical clinical applications, systematic translational barriers persist, among which data privacy and security represent fundamental challenges. Multimodal data fusion in medical AI entails patients’ sensitive imaging, pathological, genetic, and clinical information, and is subject to increasingly stringent regulatory frameworks. Traditional centralized data aggregation approaches are confronted with risks of privacy breaches and non-compliance, whereas the federated learning framework offers a viable technical avenue for large-scale multi-center data fusion by preserving raw data locally at each participating institution and only exchanging model parameters (46). Algorithmic bias and fairness issues also directly compromise the clinical credibility and ethical validity of AI systems. Disparities in the diagnostic performance of medical imaging AI across different populations have been evidenced in multiple domains (21). Patients with Sjögren’s disease exhibit marked heterogeneity in terms of gender, geography, and immunophenotype. In the absence of representative training data, the diagnostic performance of the model for specific populations will deteriorate drastically. This necessitates the concurrent implementation of mechanisms for ensuring diverse representativeness and performance monitoring during data collection and model development, as well as the establishment of a strategy for balancing diagnostic accuracy across populations. Requirements for regulatory approval and clinical evidence constitute an institutional barrier. Regulatory authorities including the US Food and Drug Administration (FDA), the European Union’s Medical Device Regulation (MDR), and China’s National Medical Products Administration (NMPA) mandate multi-center external validation and real-world effectiveness evaluation for the marketing of AI-powered medical devices. External validation studies commonly encounter the issue of performance decay (16). Accordingly, an AI-based SjD diagnostic system must demonstrate its diagnostic value via a rigorously designed prospective multi-center clinical trial, and additionally prove its contributions to pragmatic clinical endpoints such as shortening diagnostic delays and optimizing clinical decision-making. Physician acceptance and integration with clinical workflows represent another dimension of challenges. The insufficient interpretability of medical AI and difficulties in workflow integration are core factors impeding clinical adoption (24). SjD diagnosis involves multi-specialty collaboration; AI systems are required to achieve seamless integration with existing information systems, while reducing physicians’ cognitive load through interpretable decision representations. Research has indicated that models designed under the concept of physician-AI collaboration tend to yield superior clinical acceptance and diagnostic accuracy. Additionally, the issue of probability calibration in medical AI may also undermine the effectiveness of clinical decision-making (50).

In this review focusing on single-center, retrospective, small-sample, and imbalanced data scenarios, external generalizability remains a core source of uncertainty. Imaging AI commonly demonstrates performance degradation during cross-institutional validation, indicating that “domain shift” significantly impacts diagnostic/segmentation tasks. Future research should prioritize multi-center, prospective external validation as a design prerequisite and adhere to Consolidated Standards of Reporting Trials (CONSORT) and Radiological Society of North America (RSNA) guidelines in reporting to enhance overall research quality (51). Feature stability in imaging remains a bottleneck for translation: CT reconstruction kernels, slice thickness, and dose, along with MRI sequence variations and site differences, systematically alter radiomics feature distributions, limiting model robustness. Therefore, pipeline-level features and implementations should undergo Image Biomarker Standardization Initiative (IBSI) normalization and be validated through test-retest/parameter perturbation assessments. When aggregating multi-center data, batch effect corrections like ComBat should be applied alongside thorough analysis of outlier sensitivity (52). For salivary gland ultrasound, even with the OMERACT framework, operator training levels and equipment presets still affect signal consistency. It is recommended to complete operator training and parameter locking before model development, followed by external batch retest consistency before clinical evaluation (17). Regarding imaging-immunology/multi-omics integration, limited sample size, heterogeneity, and missing values pose major technical barriers. Future strategies should adopt multi-center, multimodal collaboration acquisition approaches and incorporate robust cross-modal alignment and missing value processing pipelines in research designs (37).

## 6 Future expectations

The application of artificial intelligence in Sjögren’s disease is poised to transcend its current, predominantly diagnostic role, evolving into a foundational technology that reshapes the entire patient journey from early detection to personalized therapy. To realize this potential, future efforts must converge on several pivotal fronts. First, the move beyond unimodal analysis to robust multimodal data fusion is imperative. Integrating clinical, imaging, histopathological, genomic, and metabolomic data into a unified AI model will be crucial to capture the disease’s full heterogeneity and establish a comprehensive diagnostic ecosystem (53). Second, we foresee the rise of dynamic, AI-powered monitoring systems. Leveraging data from wearables and mobile health technologies, these systems will shift the paradigm from episodic clinic visits to continuous, proactive management, enabling preemptive interventions (54). Third, and most critically, the field must advance from correlative patterns to causal models and actionable insights. This entails developing interpretable AI that can deconstruct SjD into mechanistically defined subtypes. Such a granular understanding is the prerequisite for true precision medicine. It will empower AI-driven drug repurposing and target discovery—exemplified by studies that already screen core hub genes from thousands of candidates via integrated machine learning and molecular docking (55)—to transition from in silico predictions to viable clinical candidates.

To translate these principles into tangible progress, we propose a stratified roadmap with short-, medium-, and long-term priorities. Short-term research will adopt standardized data collection protocols developed by OMERACT and the IBSI to perform prospective multicenter external validation on existing predictive models. This covers deep learning algorithms for SGUS, CT radiomics classifiers, and pathology models built on CLAM architecture. We also recommend constructing a publicly accessible benchmark dataset with complete standardized annotations. Over the medium term, research priorities will shift toward clinically viable multimodal fusion frameworks to resolve real-world diagnostic dilemmas encountered in routine care. We propose a unified fusion model capable of jointly analyzing imaging, histopathology, serology and patient-reported outcomes. This design can accurately distinguish Sjögren’s syndrome from lookalike conditions, including IgG4-related disease, sarcoidosis and chronic sialadenitis. Long-term work will shift the research framework from one-off diagnostic identification to full-cycle precision management guided by disease mechanisms. Causal graphical models can pinpoint modifiable pathogenic triggers, helping uncover druggable targets and support in silico drug repurposing. Combined wearable biosensors and AI alert systems will enable continuous community monitoring, allowing preventive care for mild or oligosymptomatic early-stage patients. Physician-led clinical validation is mandatory across every stage of model development. We advocate pragmatic clinical trials centered on patient-oriented outcomes: shorter diagnostic delays, fewer unnecessary invasive biopsies, and improved quality of life. Such evidence will help translate AI tools from theoretical research outputs into practical, essential tools for routine Sjögren’s syndrome management.

The final translational hurdle will be overcome through innovative clinical trial designs that explicitly validate these AI-derived biomarkers and therapeutic strategies. We contend that the ultimate goal is not merely to create a diagnostic assistant, but to forge an AI-coordinated clinical decision support system, capable of guiding individualized treatment plans and fundamentally improving patient outcomes.
